# Ophthalmology workforce over a decade in the Kingdom of Saudi Arabia: demographics, distribution, and future challenges

**DOI:** 10.1186/s12960-024-00902-3

**Published:** 2024-03-04

**Authors:** Tariq Aldebasi, Abdullah M. Alhejji, Bushra H. Bukhari, Nawaf K. Alawad, Sarah M. Alghaihab, Raghad M. Alakel, Albanderi Alhamzah, Tariq Almudhaiyan, Shatha Alfreihi, Malek Alrobaian, Shiji Gangadharan

**Affiliations:** 1 Department of Ophthalmology, King Abdulaziz Medical City, National Guard Health Affairs, Riyadh, Saudi Arabia; 2 King Abdullah International Medical Research Center, Riyadh, Saudi Arabia; 3 College of Medicine, King Saud Bin Abdulaziz University for Health Sciences, Riyadh, Saudi Arabia; 4 Department of Emergency Medicine, King Abdulaziz Medical City, National Health Affairs, Riyadh, Saudi Arabia; 5 Department of Surgery, Division of Ophthalmology, King Fahd University Hospital, Khobar, Saudi Arabia; 6 Department of Pediatric Surgery, Division of Pediatric Ophthalmology, National Guard Health Affairs, Riyadh, Saudi Arabia

**Keywords:** Ophthalmology, Ophthalmology workforce, Saudi Arabia

## Abstract

**Background:**

The ophthalmology workforce is an integral component of any health care system. However, the demand for eye care has imposed a heavy burden on this system. Hence, this study aimed to estimate the trends, demographic characteristics, distribution, and variation between Saudi and non-Saudi ophthalmologists and the future challenges of the ophthalmology workforce in the Kingdom of Saudi Arabia (KSA).

**Methods:**

This study was conducted in the KSA and included ophthalmologists practicing from 2010 to 2023. From the Saudi Commission for Health Specialties, we obtained the number, gender, nationality, and rank of ophthalmologists. The geographic distribution of ophthalmologists in the KSA was obtained from the Ministry of Health Statistical Yearbook 2021.

**Results:**

As of January 2023, the KSA had a total of 2608 registered ophthalmologists, with approximately 81.06 ophthalmologists per 1,000,000 people. Only 38% of all ophthalmologists in the country were Saudis. The percentage of Saudi female graduates increased from 13.3% to 37.2% over 12 years [Sen’s estimator of slope for median increase per year = 1.33 (95% CI 1.22–1.57) graduates; trend test *P* < 0.001). Additionally, we found that the geographic distribution of ophthalmologists varied (test for homogeneity of rates, *P* < 0.0001), with the larger regions having a higher concentration of ophthalmologists than the smaller regions (75.6 in Riyadh versus 42.8 in Jazan per 1,000,000 people). However, the World Health Organization's target for the ophthalmologist-to-population ratio has been achieved in all 13 health regions of KSA.

**Conclusion:**

The recommended ophthalmologist-to-population ratio has been achieved in the KSA, and the number of Saudi ophthalmologists has almost doubled over the past 8 years. However, the majority of ophthalmologists are still non-Saudi, as Saudi ophthalmologists constitute approximately one-third of the ophthalmology workforce in the KSA. The geographical distribution of ophthalmologists varies, which might affect access to care in peripheral regions. In response to the growing demand for eye care in the KSA, several more effective measures might need to be considered.

**Supplementary Information:**

The online version contains supplementary material available at 10.1186/s12960-024-00902-3.

## Introduction

Ophthalmology is a branch of medicine concerned with the diagnosis and treatment of eye diseases. Ophthalmologists play a significant role as health care providers. Specifically, they use surgical and nonsurgical means to manage eye trauma, anterior segment diseases, retinal pathologies, autoimmune disorders, infections, uveitis, strabismus and ocular pediatric pathologies, tumors, and congenital disorders [1].

The ophthalmology workforce is an integral component of any health care system. However, the demand for eye care has imposed a heavy burden on this system. Previous studies have shown the increasing magnitude of medical demand, and the growth patterns of medical demand have critical management implications for optimizing medical supply and improving the safety and quality of medical care [2]. A study performed from 1995 to 2017 showed that the national density of ophthalmologists in the United States (US) has decreased from 6.30 to 5.68 ophthalmologists per 100,000 people, and the workforce is in the older age group [3]. While the annual number of training residents has increased by 1–2%, it appears that the rate of increase in ophthalmologists practicing in the U.S. will not keep up with the rate of increase in citizens over the age of 65 [4]. Reports from similar studies are also consistent with these results [5–7]. Ophthalmology is one of the surgical specialties estimated to have a shortage of surgeons by 2030. As a result, the clinical workload is estimated to increase by 10–50% and 7–61% by 2030 and 2050, respectively [8]. Similarly, the required number of ophthalmologists in Singapore is projected to increase by 117% from 2015 to 2040 [9].

The Australian Health Practitioner Regulation Agency's dataset contains records for 1056 ophthalmologists, with the proportion of ophthalmologists working outside major cities increasing from 19% in 2014 to 24% in 2019 [10]. This is consistent with data from the U.S., where there was a mean annual increase of 2.3% in the density of ophthalmologists working in rural areas [3]. Despite the trend toward an increasing proportion of ophthalmologists working outside major cities, the workforce is still maldistributed [10]. In contrast, a recent study conducted in New Zealand revealed that the number of ophthalmologists in each region is proportional to the population [11].

In 2020, Resnikoff et al. reported the number of ophthalmologists in the Kingdom of Saudi Arabia (KSA) to be 80.7 per million people [7]. The General Authority of Statistics (GASTAT) estimated a population of 32.1 million in the KSA as of 2022 [12]. As a part of the Saudi 2030 vision, population growth is expected, and medical tourism will increase significantly in the KSA, which will increase the demand for physicians, including ophthalmologists. In developed nations, there are more practitioners than ever, but the population of people aged 60 and older is growing twice as quickly as the industry is. Thus, it is imperative to actively train eye care teams today to address the current and anticipated shortage of ophthalmologists around the world to bridge the growing gap between demand and supply. Given the data-intensive nature of this specialty, data play an important role in ophthalmology [13]. Indeed, the power of knowledge can lead to great advancements. To date, no studies have been performed in the KSA to estimate the size of the ophthalmology workforce. Therefore, there are variable opinions on whether the supply of current ophthalmologists will be adequate for accommodating the increasing population in the country. Such studies are crucial for guiding future strategies that aim to deliver a comprehensive, well-distributed eye care delivery system across the nation. Finally, this research will provide valuable information to those in authority to make necessary changes and will facilitate the decision-making process for future generations of medical students when choosing their specialty.

Our research objective is to estimate the trends, demographic characteristics, distribution, and variation between Saudi and non-Saudi ophthalmologists and to explore future workforce challenges in the field of ophthalmology in the KSA. Additionally, we aim to clarify whether there is an uneven distribution of ophthalmologists across the kingdom and to determine the ratio of ophthalmologists to the population density.

## Methodology

We performed a study of all ophthalmologists practicing in the KSA from 2010 to 2023. The Kingdom consists of 13 regions, all of which were included in the study. According to the latest census of 2022 by the GASTAT, the population of KSA was 32,175,224 [12]. Institutional review board (IRB) approval was obtained from King Abdullah International Medical Research Center, National Guard Health Affairs, Riyadh, Saudi Arabia.

To achieve the aims of this study, the Saudi Commission for Health Specialties (SCFHS) was contacted via email to provide the following data: (1) the number of ophthalmologists practicing in the KSA annually from 2010 to 2023; (2) the number of Saudi and non-Saudi ophthalmologists; (3) the number of male and female ophthalmologists; (4) the number of ophthalmologists at each level from residents to consultants (ranks and descriptions of physicians according to the SCFHS are presented in Table 1, [14]); and (5) the number of matched and graduated physicians from Saudi ophthalmology residency programs from 2010 to 2023. In addition, data about the geographic distribution of ophthalmologists practicing in the country were obtained from the Saudi Ministry of Health (MOH) Statistical Yearbook 2021 [15].

**Table 1:** Ranks of physicians in the Kingdom of Saudi Arabia according to the SCFHS

Rank	Description	
Consultant	A physician who has a Saudi specialization certificate or its equivalent with at least 3 years’ worth of experience working in a recognized hospital or health care facility	
Senior Registrar	A health care professional who has obtained a Saudi specialization certificate or its equivalent	
Registrar	A health care provider who has had a specialty certificate for at least 2 years and who has fulfilled the necessary experiences, with a minimum total training time of 4 years	
Training Resident	A medical professional who is enrolled in a postgraduate training program in the Saudi board certificate or equivalent	
Resident	A general practitioner who holds a bachelor’s degree in medicine and surgery from an accredited medical college and has fulfilled other classification requirements	

SCFH: Saudi Commission for Health Specialties

## Statistical analysis

Data were expressed as frequency (%) or rates (per 1,000,000 people). To calculate the ratio of ophthalmologists to the population, the number of ophthalmologists was divided by the entire population and then multiplied by 1,000,000. Categorical data were compared using χ2 test. As the change in rate is not expected to be linear from year to year (with no correlation between measurements collected at different times), we employed the Mann–Kendall test for Monotonic Trend (MK test) rather than parametric linear regression analysis which requires that the residuals from the fitted regression line be normally distributed; an assumption not required by the MK test. Linear trend (median increase per year in number of ophthalmologist) was assessed using the robust linear regression Theil–Sen estimator. The choice of this technique was based on the fact that it is significantly more accurate than simple linear regression for skewed and heteroskedastic data and compares well against non-robust least squares even for normally distributed data in terms of statistical power. Differences in rates by geographical area were conducted using the test for homogeneity of rates. Data analysis was conducted using IBMSPSS Statistics for Windows, version 26 (IBM Corp., Armonk, N.Y., USA).

## Results

**Fig. 1:**
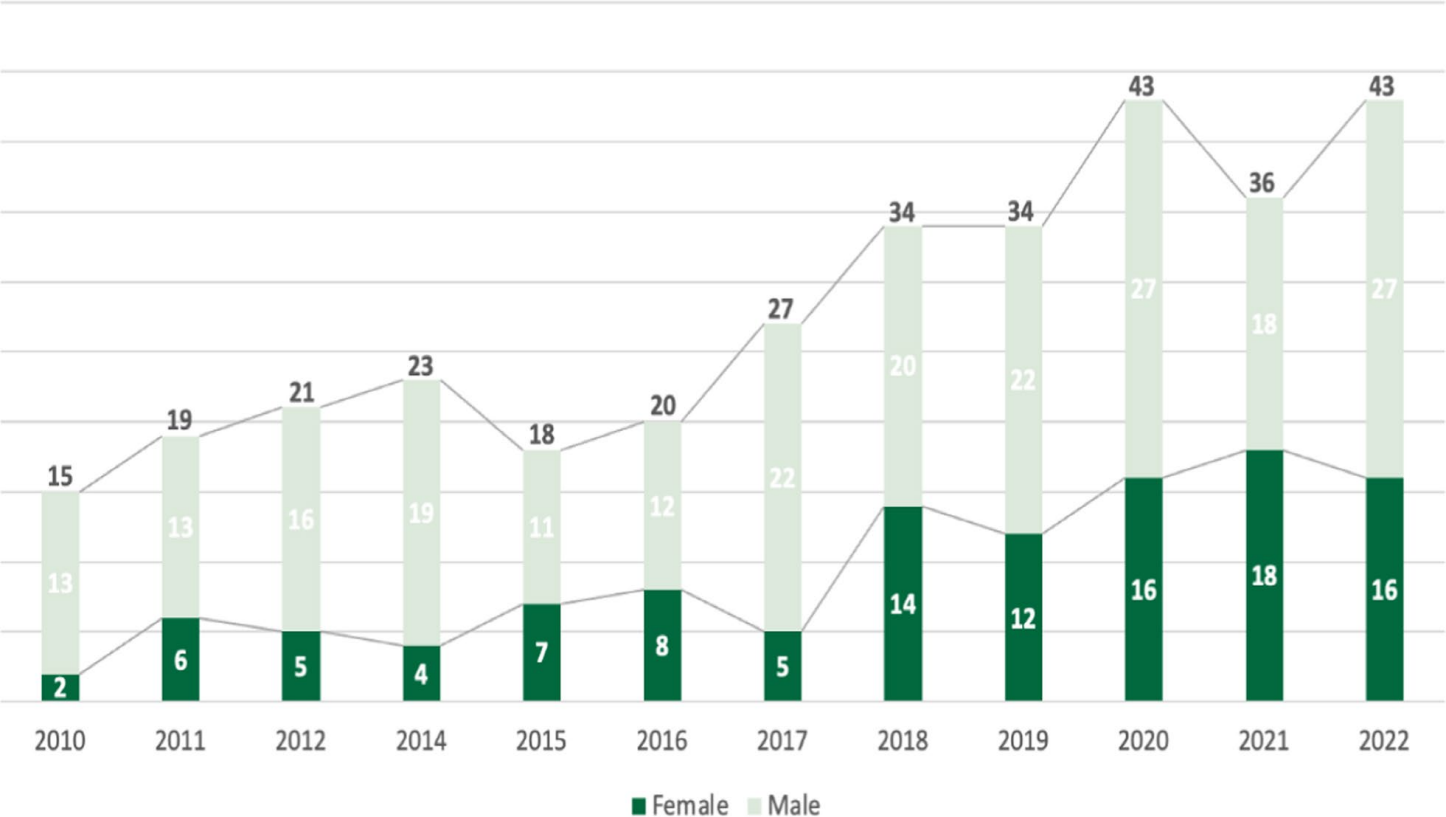
Number of Saudi female and male ophthalmology graduates by year (MK trend test: overall *p* < 0.001, males *p* < 0.01, females < 0.001)

As of January 2023, KSA had a total of 2608 registered ophthalmologists, with approximately 81.06 ophthalmologists per 1,000,000 people. Only 38% (*n* = 983) of all the ophthalmologists in the country were Saudi. Male ophthalmologists were more common among both Saudi and non-Saudi registered ophthalmologists (69.7%). Regarding the ranking of practicing ophthalmologists in the country, 37% (*n* = 972) were registrars, of which 95% were non-Saudi, and 70% were male. Consultants constituted 33% (*n* = 856) of practicing ophthalmologists, of which 65% (*n* = 557) were Saudis. Out of 239 training residents, only three were non-Saudis. There was only one training resident for approximately every four consultants. The percentage of male residents was 59% (*n* = 81). Registered number of ophthalmologists as of 2023 differ by gender for both Saudi and non-Saudi groups; *P* < 0.0001. Additional information is shown in Table 2.The data presented in Fig. 1 show the annual counts of Saudi ophthalmology graduates categorized by gender from 2010 to 2022. In 2010, the proportion of male-to-female graduates was 6.5:1. However, over 12 years, the percentage of Saudi female graduates increased from 13.3% to 37.2%, with the most significant change occurring between 2018 and 2022. In 2022, there were 16 and 27 Saudi female and male graduates, respectively, for a 1.7:1 male-to-female ratio. Overall, Sen’s estimator of slope (median increase per year) was 2.47(95% CI 2.10–3) graduates (MK trend test *P* < 0.001), for males was 1.25(95% CI 1–1.57) graduates (MK trend test *P* < 0.01) and for females was 1.33 (95% CI 1.22–1.57) graduates (MK trend test *P* < 0.001).

**Table 2:** Number of registered ophthalmologists in Saudi Arabia by rank (as of January 2023)

Rank	Saudi		*P*-value	Non-Saudi		*P*-value	Total
	Male	Female		Male	Female		
	n (%)	n (%)		n (%)	n (%)		n (%)
Consultant	423 (63.7)	134 (42)	*P* < 0.0001	258 (22.4)	41 (8.7)	*P* < 0.0001	856 (32.8)
Senior Registrar	84 (12.7)	41 (12.9)		167 (14.5)	112 (23.7)		404 (15.5)
Registrar	25 (3.8)	25 (7.8)		654 (56.7)	268 (56.8)		972 (37.3)
Training Resident	124 (18.7)	112 (35.1)		1 (0.09)	2 (0.42)		239 (9.2)
Resident	8 (1.2)	7 (2.2)		73 (6.3)	49 (10.4)		137 (5.3)
Total	664	319		1153	472		2608

Table 3 shows the number of doctors in the KSA who were matched to and graduated from an ophthalmology residency program between 2010 and 2022. Ophthalmology residency programs in the country span four years (from R1 to R4). The number of doctors who graduated from the program increased from 15 in 2010 to 43 in 2022, tripling over the 12 years. Moreover, the number of matched physicians increased by 136%. In 2018, 44 residents were matched to the program, and four years later, 43 had graduated from it.

**Table 3:** Numbers of doctors matched to and graduated from ophthalmology residency programs, Saudi Arabia

Year	Matched	Graduated	
2010	25	15	
2011	25	19	
2012	26	21	
2013	26	*	
2014	40	23	
2015	40	18	
2016	45	20	
2017	44	27	
2018	44	34	
2019	50	34	
2020	53	43	
2021	53	36	
2022	59	43	

^*^Unavailable data

**Fig. 2:**
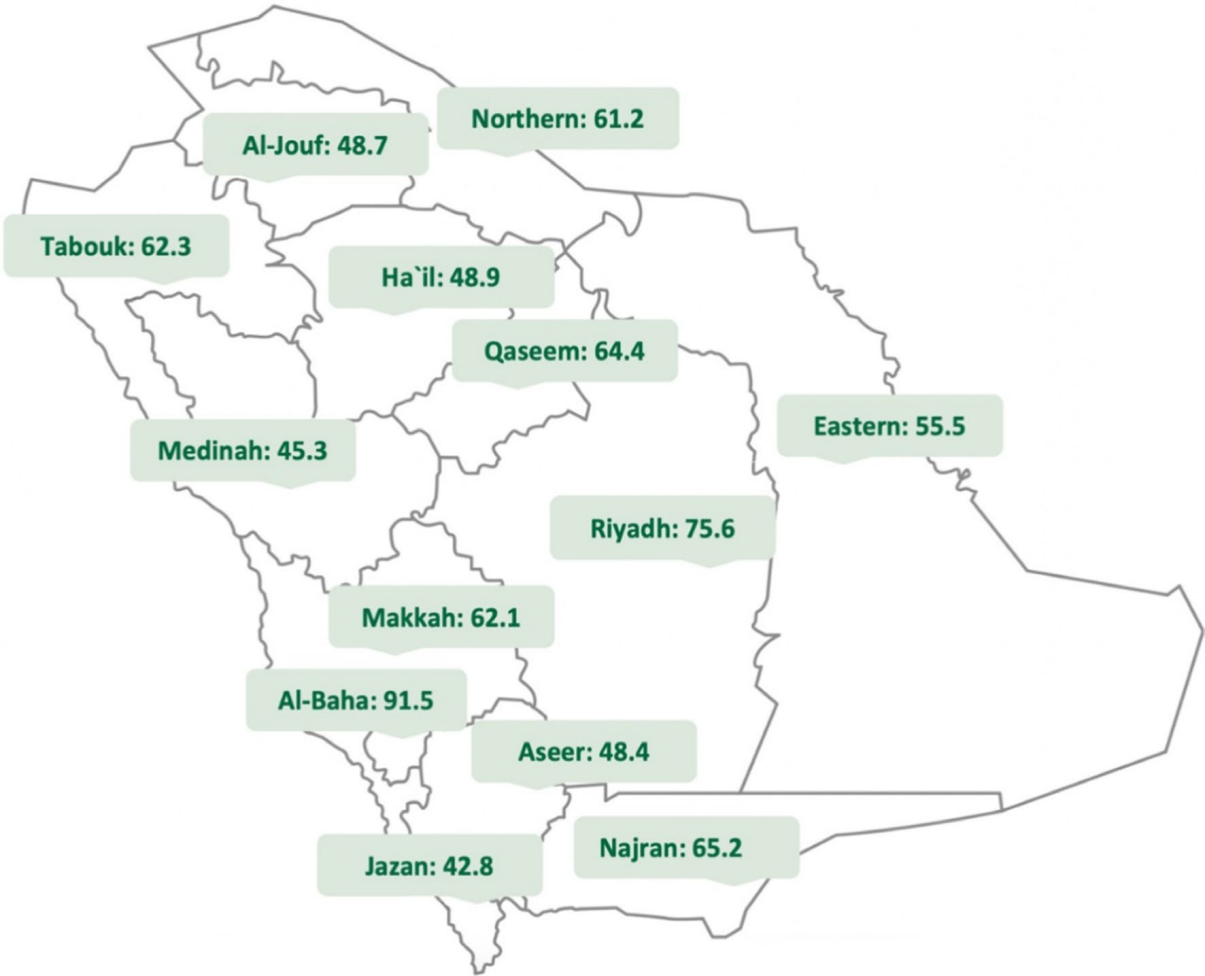
Ophthalmologists per 1,000,000 people in Saudi Arabia by geographical region, 2021 (test for homogeneity of rates, *P* < 0.0001)

In 2021, the majority (85%) of ophthalmologists practicing in the KSA were working in either Saudi MOH hospitals or the private sector, while the remaining 15% were practicing in other governmental sectors. The geographic distribution of ophthalmologists practicing in Saudi MOH hospitals and the private sector (data from other governmental sectors were unavailable, so they were excluded) in 2021 is depicted in Fig. 2, with the number of ophthalmologists ranging from 42.8 to 91.5 per 1,000,000 people and differed statistically (test for homogeneity of rates, *P* < 0.0001) across the 13 health regions of KSA. The most populated regions in the KSA are Riyadh and Makkah. The number of ophthalmologists in Riyadh was 75.6 physicians per million people, 42.2 of whom worked in the private sector. The lowest densities of ophthalmologists were 42.8, 45.3, and 48.4 per 1,000,000 people in Jazan, Medinah, and Aseer, respectively. The regions with a population lower than 1 million (Tabouk, Ha'il, Najran, Al-Jouf, Al-Bahah, and Northern Borders) had more ophthalmologists practicing in MOH hospitals than in private sectors. The Al-Baha region had 91.5 ophthalmologists per 1,000,000 people, which reflects its relatively small population size of 327,833 people. The 2021 ophthalmologist-to-population ratio was 1:13,500 in the kingdom. For more information on the ophthalmologist-to-population ratio of the 13 health regions of KSA, refer to Additional file 1: Table S1. The ophthalmologist population increased at a rate of 14.74%. While the overall population experienced an increase of 4.52%, the 60+  year-old demographic population experienced an increase of 6.12%. This led to an actual growth rate in the ophthalmologist population of 10.22% for the year 2022 for the overall population and 8.62% for the 60+ year-old population (Table 4). We also calculated ophthalmologists growth rate versus population growth rate in Saudi Arabia for the last decade. (Additional file 1: Table S2).

**Table 4:** Ophthalmologists growth rate versus population growth rate, Saudi Arabia, 2022

	All ages (%)	60 + (%)	
Ophthalmologists growth rate	+ 14.74	+ 14.74	
Population growth rate	+ 4.52	+ 6.12	
Actual growth rate	+ 10.22	+ 8.62	

## Discussion

### The number of ophthalmologists worldwide

**Fig. 3:**
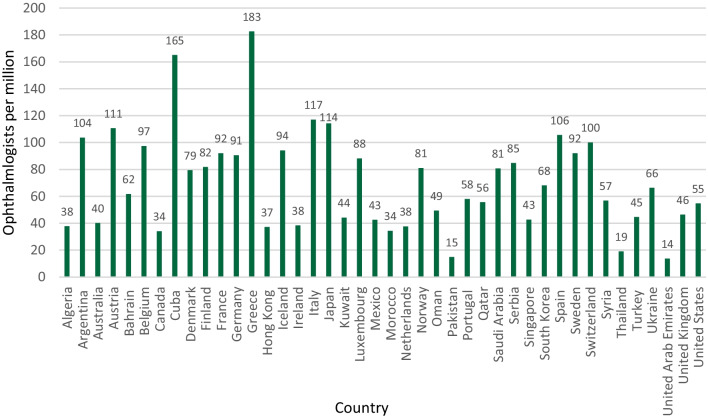
Ophthalmologists per 1000 000 people worldwide, 2015*. *Adopted from the International Council of Ophthalmology [7]

In 2010, the International Council of Ophthalmology (ICO) charted 213 national societies of ophthalmology in 193 countries to gather statistics about the worldwide demographics of ophthalmologists. There are more than 200,000 ophthalmologists worldwide, but the number of ophthalmologists in developing countries is insufficient, and the aging population is growing faster than the profession is. Although a 2015 survey revealed that the global ophthalmologist workforce has increased by 14% since 2010 and is growing at an annual rate of 2.6% (compared to the 1.2% growth estimate in 2010), this workforce rate still lags behind the growth rate of the global aging population, which is 2.9% [7]. Figure 3 shows the number of ophthalmologists per million people worldwide. Greece has the highest number of ophthalmologists (183 per million people). Within the Gulf Cooperation Council (GCC) countries, the KSA has the largest number of ophthalmologists, with a density of 80.7 practitioners per million people. Conversely, the United Arab Emirates stands at the lower end of the spectrum among the GCC nations, with a density of only 14 ophthalmologists for every million individuals. In contrast, data from the Saudi MOH annual statistical yearbook from 2015 reported a total of 1811 ophthalmologists practicing in the country, equaling approximately 57.5 ophthalmologists for every million individuals [16].

In 2020, the ophthalmology workforce consisted of approximately 18,500 ophthalmologists in the U.S. Over the last few decades, the annual growth rate of residents in training has been modest at 1–2%, which is significantly lower than that of those in the field of optometry. Moreover, fewer than 20 international residency graduates enter the U.S. workforce every year [4]. In a recent study examining workforce projections in the U.S., the supply of ophthalmologists was projected to decrease by 12%, whereas the demand for their services is anticipated to increase by 24%. This situation is likely to lead to a substantial imbalance, with a predicted 30% mismatch between supply and demand by the year 2035 [17].

### The number of ophthalmologists in Saudi Arabia

**Fig. 4:**
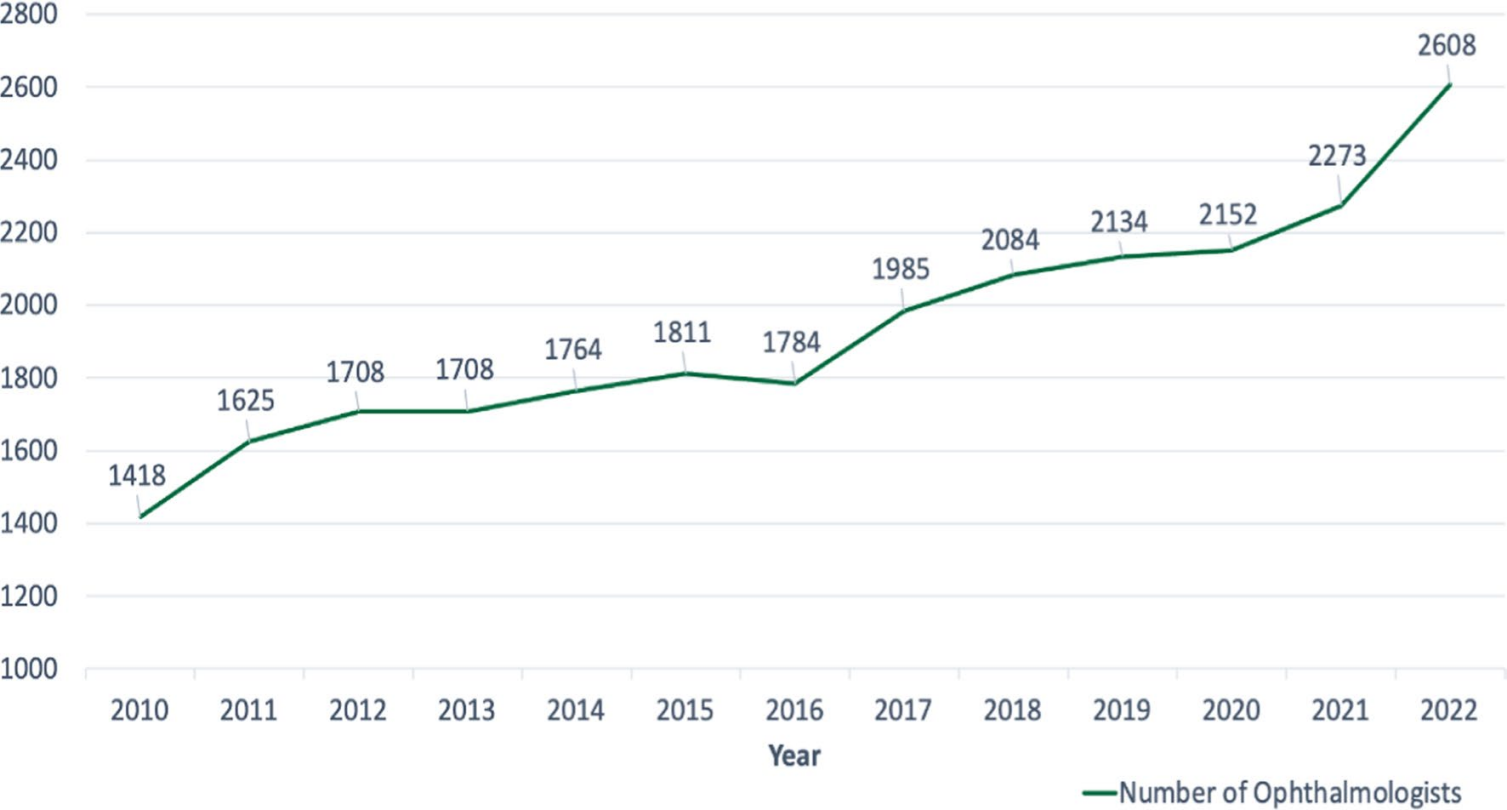
Total number of registered ophthalmologists in Saudi Arabia, 2010–2022. *Adopted from MOH statistical yearbooks

The total number of ophthalmologists practicing in the KSA almost doubled in a decade, reflecting the successful Saudi MOH initiatives (i.e., the MOH 2020 initiative in collaboration with the SCFHS under the umbrella of Saudi 2030 Vision strategies) (Fig. 4). In 2022, it was evident that the growth rate of ophthalmologists in the kingdom is outpacing both the overall population growth and the growth of the population aged 60 years old and above. By January 2023, the kingdom had almost 81.06 ophthalmologists per million people. This trend could indicate an encouraging improvement in health care provision, particularly in ophthalmic care, as the increasing number of ophthalmologists suggests an enhanced capacity to address ocular health concerns. This is especially crucial given the aging population's greater susceptibility to vision-related conditions.

### The expansion of ophthalmology programs over the last decade in Saudi Arabia

The Saudi MOH initiative was established in collaboration with the SCFHS to reinforce the total Saudi health care workforce by expanding residency training capacities and the number of annual trainees enrolled in the SCFHS programs; there was a particular interest in augmenting the number of trainees in ophthalmology residency programs since there were only 551 Saudi ophthalmologists in 2012 out of 1708 total ophthalmologists, meaning that for every million inhabitants, there were 19 Saudi and 57 total ophthalmologists [18]. The goal number of Saudi ophthalmologists was set at 2100; hence, an additional 1500 ophthalmologists were needed to reach an average of 60–70 physicians per million people, for a total of 3144 ophthalmologists by 2030, however the execution of the roadmap to close the gap is currently underway.

Compared to 2013, in which the total number of accepted trainees in the ophthalmology residency program was only 26 per year distributed across 3 programs, the substantial governmental efforts resulted in an increase of more than 127% through the addition of 33 additional candidate seats, reaching a total of 59 accepted residents in 2022 [19]. An increased number of accepted trainees was attained through expanding the existing centers in the central, eastern, and western provinces, as well as forming novel training programs around the country. Currently, there are eleven residency programs (Riyadh-5, Jeddah-1, Makkah-1, Madina-1, Taif-1, Eastern Province Joint Program-1, and Southern Province Joint Program-1) in the Kingdom. A comparison of the number of program graduates between 2015 and 2022 reveals a notable increase of 139%, with the number of graduates rising from 18 to 43. Expansion efforts have led to a significant increase in Saudi female ophthalmology graduates, which will be reflected in workforce counts. As of January 2023, the cumulative number of Saudi ophthalmology practitioners was estimated to be 983 physicians, which is nearly double the number recorded in 2012 [18]. Of these, 557 were consultants, 125 were senior registrars, 50 were registrars, and 236 were undergoing residency training at the time. In their survey, Resnikoff et al. reported 1.7 training residents per million in low-income countries, 5.7 in lower-middle-income countries, 7.8 in upper-middle-income countries, and 8.5 in high-income countries [20]. Only 3.6 ophthalmology training residents were present in Saudi Arabia for every million people in 2013, but by 2023, that number had risen to 7.3. Moreover, Saudi Arabia has been sending a significant number of training residents abroad for specialized medical training in various fields, including ophthalmology.

### Foreign ophthalmologists in Saudi Arabia

Due to the rapid industrialization in the kingdom over the last 4 decades, it was necessary to compensate for the deficit in educational infrastructure and the number of Saudi health practitioners by recruiting and employing health practitioners from abroad [21]. Despite the drastic increase in educational capacity, the majority of health care workers continue to be non-Saudi workers. In 2016, foreign doctors constituted approximately 73% of the total health workforce in the KSA, in comparison to the US, where they comprise only 27.9% [22]. Over the past decade, the percentage of foreign ophthalmologists practicing in the Kingdom has decreased from 68% to 62.3% [15]. Many challenges exist with the reliance on foreign physicians. A high rate of turnover negatively impacts health care outcomes, facility management, and staffing. Sinsky et al. concluded that primary care physician turnover results in increased annual health care costs [23]. Furthermore, foreign physicians face multiple difficulties related to cultural and language differences. In addition, poor communication leads to both physician and patient dissatisfaction and increases health care costs [24, 25]. The WHO Global Strategy on Human Resources for Health: Workforce 2030 recommended the following strategies to decrease dependency on foreign-trained health workers: increasing investment in national health professional education; aligning government educational expenditure with employment opportunities; implementing innovative financing plans; and encouraging more cost-effective ways to educate health professionals to respond to population needs [26].

### Geographic distribution of ophthalmologists in Saudi Arabia

One of the problems that affect the medical workforce worldwide is the geographic maldistribution between rural and urban areas. The WHO recommendation for the ophthalmologist-to-population ratio has been achieved in the KSA in general; however, some variation among the administrative areas still exists. Motowa et al. reported that the ophthalmologist-to-population ratio in the country in 2012 was 1:45,500 at secondary and tertiary-level government institutions [14, 27]. Another study performed in Tabuk City concluded that the ophthalmologist-to-population ratio in 2016 was 1:64,000 [28]. Our results showed that the ophthalmologist-to-population ratio was 1:13,500 in 2021. Although this ratio is higher than that in the U.S. (1:18,000), the distribution of health care providers across different regions of the KSA poses challenges. In smaller regions such as Aseer, for instance, the ratio is lower (1:21,000) [4]. More information is provided in Additional file 1: Table S1. The uneven geographical distribution of health workers complicates accessibility to health care, resulting in suboptimal outcomes and further increasing health costs. Teleophthalmology and artificial intelligence might serve as good solutions to this issue [29]. Moreover, Saudi governmental initiatives have taken proactive measures to improve geographical imbalances in ophthalmic care. This has been achieved through the establishment of new ophthalmology programs across the kingdom, reflecting a targeted effort to broaden access and distribute resources more equitably.

### Present Saudi ophthalmology workforce and future recommendations

With a growing number of ophthalmology programs and the availability of tertiary eye care centers, the aim of 8.5 residents per million can be met by 2025. However, access to eye care can remain challenging due to a lack of skilled ophthalmologists and specialist optometry at the primary level, an unclear referral system, and the scant percentage of Saudi registrars (only 5%). Many factors could influence the future practice pattern of the Saudi Board of Ophthalmology graduates  as well as their academic goals. Al-Essa et al. conducted a study on Saudi residents in 2017 and concluded that, for the majority (81%) of respondents, the primary driver of enrollment in an ophthalmology residency training program was the ability to integrate medicine and surgery. Most of the residents indicated a desire to perform refractive surgery (62%), participate in research (85%), and work part-time in the private sector (73%). The majority (81%) of the residents stated that they wished to pursue fellowship training (81%) and practice in an urban environment. The most popular specializations for fellowship training were the anterior segment (31%) and surgical retina (15%) [30]. A similar study had comparable outcomes, as shown in its findings [31]. These two studies showed the popularity of the anterior segment as a specialty of choice in fellowships among Saudi residents, followed by surgical retina subspecialty. This could lead to a shortage of other essential subspecialties. Additionally, the strong preference for urban practices may exacerbate disparities in ophthalmic care across various regions of the KSA. With an average of 50 Board-certified graduates per year and considering the number of retired Saudi consultants and senior registrars, 15 more years are needed to replace the Non-Saudis for both ranks, consultants and senior registrars.

We recommend proceeding with the ophthalmology residency programs’ expansion strategy as per the planned roadmap of the MOH 2020 initiative to expedite the Saudization of consultants and senior registrars. Moreover, encouraging investment in telemedicine and artificial intelligence and introducing an ophthalmology diploma program to address the scarcity of Saudi registrars are both potential ways to address these deficient factors. To ensure equitable ophthalmic care, proactive measures, including outreach programs, are needed to foster training in underrepresented subspecialties and encourage practice in smaller cities. Furthermore, the availability of general ophthalmologists and optometrists in primary care centers will enhance the early detection of ocular pathologies, refractive errors, and ophthalmic screening initiatives.

## Limitations

This study has several limitations. First, no data on the subspecialties workforce or on active cataract and refractive surgeons were collected for this study. Moreover, the data on age-based categories of ophthalmologists were not collected. Additionally, the study did not address the workforce data for optometrist and other allied ophthalmic personnel who are essential part of the comprehensive eye care system. Furthermore, data regarding the geographical distribution of ophthalmologists in other governmental sectors were unavailable.

## Conclusion

The recommended ophthalmologist-to-population ratio has been achieved in the KSA, and the number of Saudi ophthalmologists has almost doubled over the past 8 years. However, the majority of ophthalmologists are still non-Saudi, as Saudi ophthalmologists constitute approximately one-third of the ophthalmology workforce in the KSA. The geographical distribution of ophthalmologists varies, which might affect access to care in peripheral regions. In response to the growing demand for eye care in the KSA, several more effective measures might need to be considered.

### Supplementary Information

**Additional file 1.** **Table S1.** The ophthalmologist-to-population ratio in health regions of Saudi Arabia, 2021. **Table S2.** Ophthalmologists annual growth rate versus population growth rate, Saudi Arabia, Over a decade.

## Data Availability

The datasets used and/or analyzed during the current study were provided by SCFHS and Saudi MOH and are available from the corresponding author upon reasonable request.

## Abbreviations

KSA: Kingdom of Saudi Arabia
SCFHS: Saudi Commission for Health Specialties
MOH: Ministry of Health
WHO: World Health Organization
