# Supplementary material for: Ophthalmology workforce over a decade in the Kingdom of Saudi Arabia: demographics, distribution, and future challenges
Source: Hum Resour Health. 2024 Mar 4;22:19. doi: 10.1186/s12960-024-00902-3 (PMC10913636; doi:10.1186/s12960-024-00902-3)
Supplement: Supplementary file 1 — Additional file 1. Table S1. The ophthalmologist-to-population ratio in health regions of Saudi Arabia, 2021. Table S2. Ophthalmologists annual growth rate versus population growth rate, Saudi Arabia, Over a decade. [file 12960_2024_902_MOESM1_ESM.docx]

|  | | |  | | |  | | |  | | | | |  |
| --- | --- | --- | --- | --- | --- | --- | --- | --- | --- | --- | --- | --- | --- | --- |
| **Table S1.** The ophthalmologist-to-population ratio in health regions of Saudi Arabia, 2021 | | | | | | | | | | | | | |  |
| **Health Region** |  | **Ophthalmologists (N)** | | | |  | **Population** | | | |  | | **Ratio** |  |
| Riyadh |  | 618 | | | |  | 8175378 | | | |  | | 13229 |  |
| Makkah Province |  | 478 | | | |  | 7692188 | | | |  | | 16092 |  |
| Medinah |  | 93 | |  | |  | 2053240 | | | |  | | 22078 |  |
| Qaseem |  | 83 | |  | |  | 1289032 | | | |  | | 15531 |  |
| Eastern |  | 271 | |  | |  | 4879962 | | | |  | | 18007 |  |
| Aseer |  | 94 | |  | |  | 1943532 | | | |  | | 20676 |  |
| Tabuk |  | 53 | |  | |  | 850859 | | | |  | | 16054 |  |
| Ha`il |  | 35 | |  | |  | 715422 | | | |  | | 20441 |  |
| Northern |  | 22 | |  | |  | 359411 | | | |  | | 16337 |  |
| Jazan |  | 58 | |  | |  | 1355099 | | | |  | | 23364 |  |
| Najran |  | 37 | |  | |  | 567533 | | | |  | | 15339 |  |
| Al-Bahah |  | 30 | |  | |  | 327833 | | | |  | | 10928 |  |
| Al-Jouf |  | 28 | |  | |  | 574894 | | | |  | | 20532 |  |
| Total |  | 2273 | |  | |  | 30784383 | | | |  | | 13544 |  |
|  |  |  | |  |  | |  |  | |  | |  | | z |

| **Table S2.** Ophthalmologists growth rate versus population growth rate, Saudi Arabia, Over a decade | | | | |  |
| --- | --- | --- | --- | --- | --- |
|  |  | **All ages (%)** |  | **60+ (%)** |  |
| Ophthalmologists growth rate |  | +5.34 |  | +5.34 |  |
| Population growth rate |  | +2.52 |  | +6.42 |  |
| Actual growth rate |  | +2.82 |  | -1.08 |  |
